# Visual Features for Improving Endoscopic Bleeding Detection Using Convolutional Neural Networks

**DOI:** 10.3390/s23249717

**Published:** 2023-12-08

**Authors:** Adam Brzeski, Tomasz Dziubich, Henryk Krawczyk

**Affiliations:** Faculty of Electronics, Telecommunications and Informatics, Gdańsk University of Technology, 80-233 Gdańsk, Poland; tomasz.dziubich@pg.edu.pl (T.D.); hkrawk@pg.edu.pl (H.K.)

**Keywords:** machine learning, artificial intelligence, computer vision algorithms, convolutional neural networks, medical image analysis

## Abstract

The presented paper investigates the problem of endoscopic bleeding detection in endoscopic videos in the form of a binary image classification task. A set of definitions of high-level visual features of endoscopic bleeding is introduced, which incorporates domain knowledge from the field. The high-level features are coupled with respective feature descriptors, enabling automatic capture of the features using image processing methods. Each of the proposed feature descriptors outputs a feature activation map in the form of a grayscale image. Acquired feature maps can be appended in a straightforward way to the original color channels of the input image and passed to the input of a convolutional neural network during the training and inference steps. An experimental evaluation is conducted to compare the classification ROC AUC of feature-extended convolutional neural network models with baseline models using regular color image inputs. The advantage of feature-extended models is demonstrated for the Resnet and VGG convolutional neural network architectures.

## 1. Introduction

Medical imaging is one of the key achievements of modern medicine, enabling countless lives to be saved every year [1]. Imaging devices can not only provide an accurate early diagnosis of severe illnesses, and is often the only way of getting one, but can also help to choose appropriate treatment paths, verify treatment efficacy, and guide surgical interventions. Along with advances in imaging techniques, computer vision algorithms are being researched to support radiologists and other physicians in the task of reading and interpreting medical images. Automatic image analysis can also provide a potential solution to essential challenges that medicine faces: interpretation errors that occur during image reading and the growing shortage of medical professionals capable of reading the images.

An important medical imaging field that attracts the attention of many researchers is endoscopy of the human gastrointestinal tract. In modern medicine, endoscopy is still the gold standard in the diagnosis of many diseases of the digestive system, including lethal cancer diseases. It enables the physician to examine the interior of organs and diagnose the early stages of a disease, providing a high chance of successful treatment. Further motivation for research in this field appeared with the introduction of wireless capsule endoscopy (WCE) in 2001, which is less invasive than traditional endoscopy and can potentially acquire images from any part of the gastrointestinal tract.

For both wireless and traditional endoscopy, an important part of the examination is the detection of any occurrence of blood in the gastrointestinal tract, resulting from both active and nonactive bleeding. The diagnostic process requires the cause of any bleeding discovered during the endoscopic examination to be established. Computer vision algorithms are being developed to offer assistance to doctors by providing methods for the automatic detection of bleeding. The task, however, is definitely challenging. Bleeding occurrences are not always clear, and many cannot be recognized by an observer without medical training. Moreover, multiple forms of noise tend to occur in endoscopic images, from natural findings, such as digestive fluid, food debris, other fluids, or bubbles, to technical difficulties that result in blurry images or light-related distortions.

Although many successful algorithms have already been presented for endoscopic bleeding detection, the task remains challenging, and research is constantly being conducted to further improve the accuracy of automatic bleeding detection methods. Current approaches to endoscopic bleeding detection include computer vision methods involving rich feature descriptors, classical machine learning methods that use well-established classifiers, and deep learning methods, based entirely on neural network training in terms of both feature extraction and the classification process. Both approaches have clear benefits and limitations.

This paper introduces a novel method that combines modern convolutional neural network architectures with dedicated high-level features to enhance accuracy in bleeding detection in a scenario of a limited training dataset size, by leveraging insights from the analysis of the blood identification task.

The paper is structured as follows. In Section 2, a review of the literature is presented. The next sections include a detailed description of the proposed method, the theoretical definition of the identified high-level features, the process of implementing the feature descriptors capturing the features in the form of computer vision algorithms, and the scheme for combining the features with deep neural network models (Section 3), and the CNN (convolutional neural network) model training procedure (Section 4). Subsequently, in Section 5, an experimental verification of the proposed approach is presented by evaluating the impact of extending the models with the proposed high-level features. Finally, a discussion and final remarks are presented in Section 6.

## 2. Existing Work

The literature analysis highlights that current bleeding detection methods fall primarily into three categories:

- Classical computer vision techniques that rely on image processing and statistical features extracted from images.
- Classical machine learning approaches employing well-established algorithms such as SVM, Random Forest, k-NN, PCA, etc.
- Deep learning approaches that apply existing convolutional neural network models to endoscopic data.

All three approaches tend to be generic, often employing commonly used image processing techniques that have been successful in various computer vision tasks. However, most of these methods lack interpretability and explainability, which applies both to intermediate results of low-level statistical features and to the operation of deep neural networks.

A comprehensive review of algorithms dedicated to the detection of various pathologies in endoscopic videos, including bleeding, was presented by Liedlgruber and Uhl [2]. Another review of methods for WCE images taking into account the occurrence of hemorrhages and blood was authored by Musha et al. [3]. A comprehensive overview of advances regarding upper gastrointestinal tract lesions was also recently presented by Vania et al. [4]. Several systematic reviews have also been published in the area of deep learning approaches: Du et al. [5], Soffer et al. [6], Piccirelli et al. [7], and Trasolini and Byrne [8].

### 2.1. Classical Computer Vision Approach

A wide range of approaches for the detection of bleeding, as well as for other endoscopic findings and diseases, have been developed on the basis of classic computer vision methods. Approaches of this kind utilize feature descriptors based on image processing and statistical measures, designed to capture specific features of the objects to be detected and represent them in the form of a feature vector, which is then classified using simple machine learning methods, e.g., SVM classifiers or shallow neural networks.

Maroulis [9] examined colorectal lesions by first applying a 2D discrete wavelet transform to grayscale images. Co-occurrence matrices were then derived from the resulting LH, HL, and HH components, and various statistical measures were computed, including energy, angular second moment, correlation, inverse difference moment, and entropy. The classification was accomplished by employing a neural network. Magoulas [10] examined tumors using the same algorithm but applied it to 16 × 16 pixel blocks instead of the entire image. Kodogiannis [11] detected abnormalities by processing RGB and HSV color planes with the NTU transform. For each plane, nine statistics were computed, including standard deviation, variance, skewness, kurtosis, entropy, energy, inverse difference moment, contrast, and covariance. Classification was performed using an adaptive fuzzy logic system. In [12], Li investigated tumors by processing RGB planes with a 2D discrete wavelet transform. A Local Binary Pattern (LBP) transformation was applied to each of the resulting LH, HL, and HH components, and 10-bin histograms were generated. Classification was performed using support vector machines (SVMs) with an RBF kernel. Yuan and Meng [13] achieved a bleeding detection accuracy of 95% with a sensitivity of 98% and a specificity of 93% using a saliency map in conjunction with an SVM on images of size 256 × 256. Zhao [14] analyzed polyps using several types of features. For color features, the image was converted into a proposed opponent color space, and a normalized 2D histogram was computed, followed by proposed color moment invariants, which were claimed to be robust against illumination, affine transformations, and blur. The texture features were extracted using the contourlet transform, LBP, and LLE algorithm. Classification was accomplished using an SVM classifier. Kumar [15] examined Crohn’s disease and extracted features using MPEG-7 descriptors. Edge histogram descriptors were used for edge features (in 16 image blocks), dominant color descriptors for color features, and homogeneous texture descriptors for texture features. Classification was performed using an SVM classifier.

In the classical computer vision approaches, most attention is paid to the feature descriptors. In the context of endoscopic bleeding detection, most methods address the color features of the images, which is strongly justified by the nature of the typical appearance of blood. Other types of features, including texture, contours, or the use of segmentation techniques are utilized less often. Feature descriptors often take the form of complex mathematical transforms that capture specific statistics of the image, which can be considered as low-level image features.

Classical approaches delivered fairly successful results in the area of endoscopic bleeding detection, especially at a time when only small datasets of endoscopic images were available. The big advantage of the classic approach was, in fact, the possibility of developing algorithms even on the basis of small collections of images. However, classic approaches face several difficulties. The development of hand-crafted feature descriptors is typically a hard and time-consuming process. The adequacy of the low-level features with the characteristic visual properties of the detected object is a challenging problem and has to be solved for every new detected object. The high complexity of the computation in the evaluation of the features, as well as the nature of common classifiers, make it difficult for a human to verify the intermediate results and the entire process. This often eliminates the possibility of identifying the reasons for the wrong predictions generated by the system. Therefore, it is difficult to include new features to improve the accuracy of an algorithm.

### 2.2. Classical Machine Learning Approach

A variety of methods for endoscopic image analysis were developed based on classical machine learning algorithms, where more emphasis is placed on employing classification methods and techniques to facilitate their training. Popular classifiers include shallow artificial neural networks, support vector machines, decision trees, and k-nearest neighbor classifiers.

Giritharan et al. [16] employed a set of support vector machine classifiers together with training data balancing based on oversampling and preprocessing using an averaging filter to remove random noise. Khun et al. [17] combined an artificial neural network with a support vector machine for color features by dividing the image into nine non-overlapping blocks. Poh et al. [18] employed an artificial neural network classifier for each cell and each block, merging the results using rule-based decision making and dividing the image into 4 × 4 cells. Yeh et al. [19] combined a C4.5 decision tree, multilayer perceptron (NLP), and a support vector machine, with feature selection using SVM feature elimination, OneR, and RELIEF algorithms. Szczypinski et al. [20] used a support vector machine classifier with an RBF kernel and reduced an extensive feature set using analysis of variance (ANOVA), sequential floating forward selection (SFFS), and the vector-supported convex hull method (VSCH). The image was divided into overlapping, circular regions. Wang and Yang [21] proposed a Bayesian classifier in which feature vectors are classified by estimating their probability of belonging to each class using the Bayesian formula and selecting the maximum score. The formula incorporates posterior probabilities for each class, derived by estimating the training dataset through the multivariate Gaussian probability density function.

### 2.3. Deep Learning Approach

Deep learning is a modern area of machine learning focused on training deep neural network models using large collections of data. Neural networks are inspired by the structure and function of the human brain, where interconnected layers of artificial neurons, organized into networks, learn to recognize patterns and make decisions. Commonly used neural network architectures are convolutional neural networks (CNNs), recurrent neural networks (RNNs), and Transformers.

The introduction of deep learning models, especially in the form of convolutional neural networks, offered a completely different approach to computer vision tasks. The feature extraction step is taken over by neural networks, which have the ability to learn features directly from images, eliminating the need for manual design and implementation. This leads to a very simple and elegant approach, where all of the processing steps, from the feature extraction to the classification step, are conducted by a single mechanism in the form of a neural network.

The rapidly growing popularity of convolutional neural networks led to the development of a variety of neural network architectures that consequently improved the accuracy for multiple computer vision tasks. For the field of endoscopic bleeding detection, a series of methods based on deep learning models was also proposed.

The majority of deep-learning-based approaches, including the work of Li et al. [22,23], Tsuboi et al. [24], Aoki et al. [25], Caroppo [26], Ghosh [27], Saraiva et al. [28], and Garbaz et al. [29], rely on existing CNN architectures including VGG [30], Resnet [31], Inception [32], and SSD networks [33] and their direct utilization for training on an endoscopic dataset of annotated blood images. Some of the approaches acknowledge the problem of a limited training data size, which is addressed by applying a transfer learning technique, typically using models trained on the Imagenet dataset [34].

A notable approach to endoscopic bleeding detection was presented by Kim et al. [35]. Although the authors only utilized an existing CNN architecture as their classification method (InceptionResnetV2 architecture), the presented approach used an outstandingly large dataset including 656,591 endoscopic images (out of which 164,713 were blood images), which enabled them to achieve high classification accuracy for multiple endoscopic findings, including bleeding. Moreover, 256,591 of the images were treated as unseen images and used by the authors in order to perform external validation of the acquired model. The localization capabilities of the model were also presented by applying class activation maps [36].

In the context of this paper, an interesting approach was proposed by Jia and Meng [37]. The authors proposed a method of combining CNN networks with hand-crafted features of blood regions. However, the hand-crafted features proposed by Jia and Meng are relatively simple low-level features that include only a histogram obtained from k-means-clustered CIE Lab color space. The features are represented as a feature vector of 50 values and are eventually concatenated with CNN activation before the classification layer. In contrast, the approach proposed in this paper uses high-level features that produce a more expressive output in the form of spatial feature activation maps. Furthermore, by passing the feature maps to the input of a CNN network (see Section 3.3), the proposed approach allows the neural network to utilize the content of the feature maps during training and prediction, while in [37], only the last classification layer can access the information contained in the features. Moreover, Jia and Meng utilized only a very small, eight-layer CNN network.

Analysis of the literature therefore shows that multiple, yet relatively simple, bleeding detection algorithms based on deep learning models have been proposed. Some of them also focus on addressing the problem of low training dataset size. Importantly, however, none of the approaches presented utilize a method equivalent to the method proposed in this work, which involves a set of high-level features dedicated for bleeding detection based on domain knowledge.

The availability of deep learning models enabled a great improvement in terms of the accuracy of computer vision algorithms. However, they also introduced a major difficulty related to the requirement of a very large training dataset size. Meanwhile, the size of available endoscopic image datasets is still not sufficient, and even though techniques including transfer learning are utilized, the application of deep learning models remains limited in the area of endoscopic bleeding detection.

## 3. The Proposed Approach

**Figure 1:**
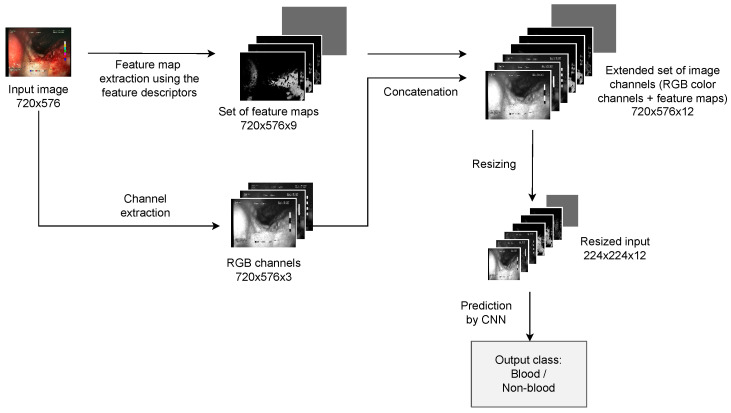
Illustration of the proposed approach including combining feature channels with a convolutional neural network. RGB color input image of 720 × 576 resolution is expected (images of different resolutions are resized, respectively). The input image is processed separately by the 9 proposed feature descriptors, each producing a 2D single-channel feature activation map (720 × 576 resolution). All feature maps combined form a tensor of 720 × 576 × 9 size, which is concatenated with original RGB channels (720 × 576 × 3), resulting in a combined output of a size of 720 × 576 × 12. The spatial resolution of the resulting tensor is finally rescaled to a size of 224 × 224 × 12 to match the typical input size of CNNs and passed as input to the model to perform the prediction (or a training step).

In order to partially address the challenges presented in the previous section, a new approach is proposed, including a combination of deep learning models and a set of proposed hand-crafted high-level visual features. The proposed features have the form of 2D feature maps, which enables them to be appended to the regular color channels of the input image as additional image channels, and therefore, for them to be passed to a regular convolutional neural network to perform the actual classification. An illustration of the proposed approach is presented in Figure 1.

The purpose of the proposed features is to provide initial clues regarding the visual characteristics of the blood to the neural network, which is expected to simplify the task of recognizing blood using the deep learning model, especially in a scenario with a limited training dataset.

The proposed approach is an extension for deep learning models that can potentially be applied in combination with other techniques to improve model accuracy, such as transfer learning or image augmentation. Therefore, it can be incorporated into deep learning approaches as another improvement to achieve greater classification performance gain.

### 3.1. Visual Features’ Definitions

The proposed high-level features were designed based on domain knowledge, as well as on the analysis of the literature in the field. The identification of the features was an iterative process including multiple proposals of candidate feature sets, assessments of their quality, and adding improvements. The design of the features was discussed with an experienced gastroenterologist in terms of the validity of the visual properties of blood that are assumed to be captured by the proposed features.

As a result of the design process, nine high-level features (denoted F1–F9) were defined. The features are proposed with simple definitions that also include exemplary images, presented in Table 1. For each image, a blue annotation is presented, illustrating the image regions where the feature is hypothetically present.

### 3.2. Feature Descriptors’ Implementation

**Figure 2:**
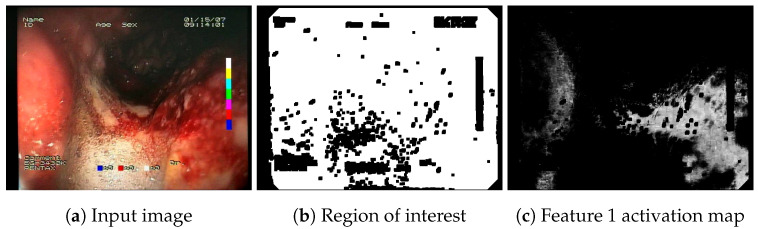
Example of a feature activation map generated by the F1 feature descriptor, along with the automatically generated ROI (region of interest) mask.

For each of the proposed features, a feature descriptor was implemented with the objective of matching definitions of the particular feature as closely as possible. Each feature descriptor was implemented in the form of a simple computer vision algorithm that transforms the input color image into a feature activation map in the form of a single-channel 2D image matching the size of the original image, where pixel intensities of the activation map reflect the strength of a given feature’s appearance in the respective part of the image. The feature descriptors were designed with an assumption of using fairly simple implementations and with a focus on vectorized image processing operations (enabling efficient execution on multicore systems). The implementations were designed using array processing methods dedicated for image processing in Python, using OpenCV and Numpy libraries. The algorithms were also tuned in terms of the choice of image processing operations and their parameters (such as kernel sizes, normalization ranges, etc.) using a novel computer-vision-oriented support tool, CVLab https://github.com/cvlab-ai/cvlab (accessed on 7 December 2023). An example of a pseudocode for the implementation of a feature descriptor (descriptor F1) is presented in Algorithm 1. The source code for the implementation of the feature descriptors is available on Github https://github.com/ambrzeski/endoblood-features (accessed on 7 December 2023). An example of a feature activation map generated by the F1 descriptor is presented in Figure 2.

### 3.3. Combining the Features with CNNs

**Figure 3:**
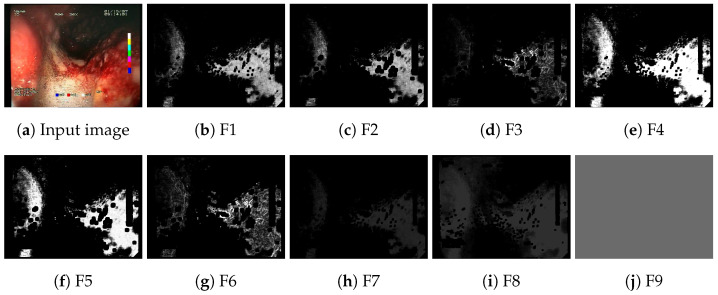
Feature activation maps generated for a sample image.

The implemented feature descriptors generate nine single-channel feature activation maps for a given input image. An example of a set of generated feature maps is presented in Figure 3. The feature maps are subsequently appended to the regular R, G, B channels of the original image, forming a 12-channel image in place of the typical 3-channel input image. The outline of the process of appending feature maps to the input of the neural network is presented in Figure 1. The resulting 12-channel image is passed as input to a regular convolutional neural network (CNN). The only modification required in the CNN architecture is the change in input size. This results only in a change in the depth dimension of the convolutional filters of the first layer of the neural network, which is modified from 3 to 12. All the remaining layers of the network remain unmodified.

The process of appending feature activation maps is identical for both model training and inference. During training of the model, for each training image, the feature maps are generated and appended to the image before passing to the network as a training data point. Similarly, in the model inference process, the feature maps need to be generated and appended to each input image.
**Algorithm 1:** Pseudocode for the *blood color region* feature descriptor (F1). RGB color channels are first extracted as *r*, *g*, and *b* arrays. Next, two coefficients are introduced to reflect color values typical for bleeding. The first coefficient ($c_{1}$) measures the excess of red component intensities over the blue and green components by measuring the minimum value of the r/b and r/g ratios, which is similar to the approach presented by Fu et al. [38,39]. The second coefficient ($c_{2}$) measures the proximity of the blue and green components, which also appears to be typical for blood regions. The two coefficients are combined by pixel-wise multiplication with weights applied (power of 5 applied to $c_{2}$). Finally, pixels with a low red component are cleared from the activation, as well as the area outside the detected region of interest. All parameters of the algorithm, including the $c_{1}$ and feature_map norm arguments, $c_{2}$ factor (power of 5), were manually tuned using the CVLab tool.1:**function** Descriptor_F1(Array img, Array roi)2:    Array *r*, *g*, *b*← extract_channels_bytes(img)3:    Array *g*← max(*g*, 1)4:    Array *b*← max(*b*, 1)5:    6:    Array $c_{1}$← min($\frac{r}{b}-1$, $\frac{r}{g}-1$)/2557:    $c_{1}$← max($c_{1}$, 0)8:    $c_{1}$← norm($c_{1}$, min=0.003, max=0.01)9:    10:    Array $c_{2}$← 1 - abs(*b* - *g*)/25511:    12:    Array feature_map←$c_{1}\cdot {\left(c_{2}\right)}^{5}$13:    feature_map← norm(feature_map, min=0.02, max=1.1)14:    feature_map[r<30]← 015:    feature_map[roi==0]← 016:    17:    **return** feature_map

## 4. CNN Model Training

Model training was conducted on a set of four neural network architectures:

- VGG19 [30]—uses a simple structure consisting of only 3 × 3 convolutions, pooling, and fully connected layers.
- Resnet50 [31]—uses the concepts of residual connections and bottlenecks.
- Resnet152 [31]—a larger variant of Resnet50 architecture.
- Inception-v3 [32]—uses the concepts of grouped and factorized convolutions.

The choice of architectures was based on their high accuracy achieved in various computer vision tasks, which has also resulted in their high popularity in both research and commercial projects [40], as well as bleeding detection algorithms (see Section 2.1). The four architectures originally gained widespread recognition after their successful application in notable ILSVRC [41] challenges. The architectures are still widely used, although they tend to be extended with additional improvements, such as the squeeze and excitation module [42], especially in the case of Resnet networks. Mixtures of these architectures are also presented in the literature, e.g., Inception-Resnet [43]. The selected convolutional neural network architectures also differ significantly from each other, not only in their size, but also as they utilize different conceptions of layers to form the architecture (except the Resnet50 and Resnet152 pair, which differ mostly in the number of layers).

For each of the architectures, a collection of models was trained in a feature-extended variant and a baseline variant that does not utilize the proposed features, to enable comparison between the variants and assessment of the impact of the proposed features.

### 4.1. Training Procedure

Training of the baseline and feature-extended models was performed using five-fold cross-validation on the training part of the dataset (details in Section 4.2). For each of the folds, a series of models was trained along with the hyperparameters’ optimization process. The optimization process was designed to find optimal values for a set of important training parameters, including learning rate, momentum, learning rate decay, batch size, and balancing the proportion of positive and negative samples, to acquire the highest possible accuracy of the model.

Separate optimizations were performed for the baseline and feature-extended models, following identical protocols and for an identical number of iterations. For both types of models, the process was conducted in two stages. The first stage included a broad hyperparameter search, in which 30 separate models per fold were trained for 5 epochs using random values of hyperparameters drawn from specified parameter ranges. In the second stage, the top six models per fold from stage one were selected and used as starting points for a set of 18 final models per fold trained in stage two training, where the training was continued until the stop condition, set to 20 epochs, without validation loss improvement.

Training was performed using the SGD (Stochastic Gradient Descent) with a momentum optimizer [44]. The learning rate was reduced in the validation loss plateau. The training batches were populated with positive and negative samples in a balanced manner following the ratio set by a hyperparameter. The experiments were carried out using Keras framework with TensorFlow backend. The training was conducted on 4 Nvidia Titan Xp GPUs.

### 4.2. Dataset

The dataset used in the experiments was collected and annotated by physicians at the Medical University of Gdańsk within the ERS project [45]. The images had been acquired with several types of analog endoscopes, digitized with a video capture device, and stored in a PAL resolution of 720 × 576 with interlacing. The recordings were captured during both gastroscopy and colonoscopy examinations. During the annotation process, a medical doctor reviewed the examinations and annotated samples in the form of short video clips. Multiple types of lesions were annotated in the project, including bleeding. In the context of blood, each sample was assigned to one of two possible classes: blood (presenting blood), considered as positive, or non-blood (no blood presence), considered as negative. More than one sample could be annotated in each examination.

The overall dataset size is 54,130 images, with 29,457 blood images and 24,673 non-blood images presenting an organ without a blood presence. Video frames were extracted from 517 different samples annotated over 372 endoscopic examinations. The dataset was split into training and test subsets using a 3/1 ratio. The statistics of the subsets are presented in Table 2. The training set was later split into five random folds to perform the cross-validation procedure, ensuring that all images from any given examination were assigned to the same fold.

#### 4.2.1. Dataset Balancing

It is a desired feature of the dataset to contain approximately balanced positive samples (presenting blood) in terms of the number of images. This would make samples similarly important during the training process. Imbalanced samples, in turn, where some blood cases include significantly more images than other cases, will have a significantly greater impact on the training and validation of the model, resulting in a strong bias towards positive samples of a large size. Unfortunately, the presented dataset suffers from a strong imbalance in the number of images per sample: almost 30% of the samples are represented just by a single image, while more than 10% of samples include more than thousand images. A slightly lower imbalance can be observed in non-blood images.

To address this issue, balancing of the samples in the blood class was applied by duplicating images in the small samples so that each sample contained at least 100 images. In this way, the disproportion in significance between the samples was reduced. Balancing was not performed, however, for the non-blood class samples.

#### 4.2.2. Data Augmentation

For the purpose of the training of the model, the images were also subjected to a set of augmentation transformations, including rotation, flip, skew, and perspective transformations, and blur, noise, and color variance transformations: hue distortion, saturation distortion, and PCA color augmentation. Following the common practice used in the field of machine learning, the magnitude of each transformation was randomly selected from predefined value ranges during each execution of the augmentation process.

## 5. Evaluation

Models trained for VGG19, Resnet50, Resnet152, and Inception v3 architectures were evaluated in order to compare the classification performance of the standard baseline neural network architecture operating on the standard RGB (red, green, blue) channels with the same network operating on the proposed feature channels appended to the RGB channels.

The evaluation was carried out in four scenarios designed to measure different aspects of model accuracy, the capability of localizing blood areas, and computation performance, and is presented in detail in Section 5.1–Section 5.4. The accuracies of models using different architectures and variants (baseline or feature-extended) are compared in a set of predefined model configurations including ensembles of groups of models. Model ensembling is performed by averaging the outputs of the models included in the ensemble. The four evaluation scenarios are presented below.

1. Best single ensemble comparison—the best models for both baseline and feature-extended variants are selected and compared. The best single ensemble (denoted as #1) is an ensemble of best-performing models from the five-fold cross-validation training; one (top) model per each cross-validation fold is used. The results are presented in Section 5.1.
2. Statistical analysis of results for multiple single ensembles—for each variant a set of 15 single ensembles (denoted as #1-15) is included and a statistical analysis is performed comparing the mean accuracy metrics values achieved by the ensembles. The results are presented in Section 5.2.
3. Blood area localization results—the abilities of both the baseline and feature-extended models to indicate of the location of the actual blood area are evaluated against reference bounding boxes that are available for part of the test set. The results are presented in Section 5.3.
4. Processing performance—an evaluation and comparison of processing performance is conducted for the baseline and feature-extended models, including the computational overheads of the features. The results are presented in Section 5.4.

### 5.1. Best Single Ensemble Evaluation

In the first evaluation scenario, the best single ensembles (denoted as #1) acquired for each variant are evaluated and compared. The best single ensemble for a given variant is an ensemble of the five best-performing single models acquired for five folds of the five-fold cross-validation training, with one (top) model for each fold. The performances of the best baseline ensembles and the best feature-extended ensembles were evaluated using a set of metrics presented in Table 3; additional definitions are presented in Table 4.

**Figure 4:**
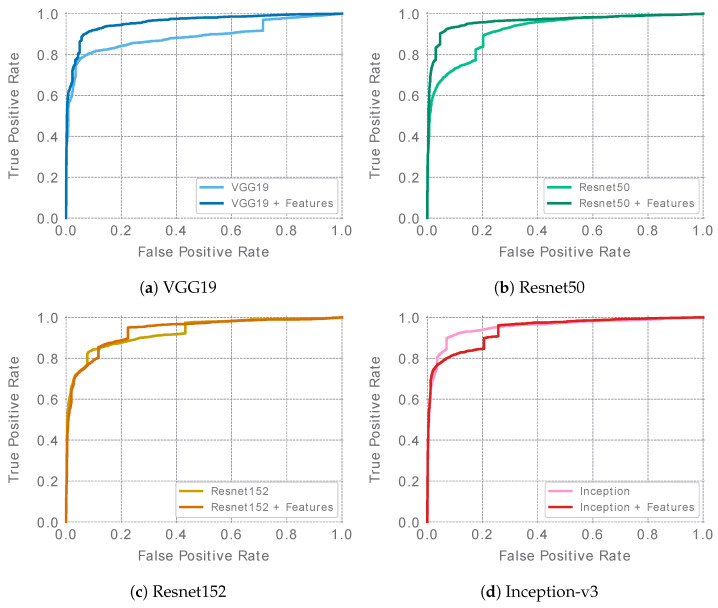
ROC curves of the best single ensembles (ensembles of 5 models—one per cross-validation fold) for each of the evaluated architectures.

The best single ensemble results for each of the evaluated architectures are presented in Table 5. ROC curves are presented in Figure 4. In the case of the Resnet and VGG19 networks, feature-extended ensembles (denoted as “+F” ensembles) achieved higher ROC AUC values than the baseline ensembles. For the Inception v3 architecture, in turn, the baseline ensemble reached a slightly higher ROC AUC. The overall accuracy considering the remaining metrics appears to be similar for the baseline and feature-extended ensembles in the case of the VGG19, Resnet152, and Inception-v3 architectures. However, in the case of Resnet50, a clear advantage of the feature-extended ensemble was observed. It was also the feature-extended Resnet50 that achieved the highest ROC AUC score of all of the best ensembles, reaching a value of 0.963.

High-sensitivity and high-specificity operating points are interesting modes for the potential production application of the models. In particular, two application scenarios of bleeding detection models can be considered. The first scenario is video summarization, where the task is to detect and filter out normal frames while preserving frames containing blood occurrences, therefore resulting in a short summary of an endoscopic video. This scenario requires the model to operate in a strictly high-sensitivity mode. The second potential scenario is a concise (but not necessarily complete) indication of the pathologies detected in the video. Such concise detection results can be appended to a preview of a video in the user interface of the imaging software. Even when not all of the lesions are presented in the detection, some of them could be cases that would be unintentionally missed during the review of the video, so presentation of the lesions decreases the chance of overlooking them. The concise form takes up little space and uses little time to be read by the physician, provided that they do not include irrelevant images that do not include any lesions. Therefore, this scenario prefers high-specificity (low false positive rate) operating points.

The high-sensitivity (spec@0.95,spec@0.99,spec@0.999) and high-specificity operating points’ (sens@0.01,sens@0.001,sens@0.0001) (see Table 3) results have already been presented in Table 5. For the sensitivity=0.95 operating point, the Resnet50+F model ensemble achieved 0.855 specificity, which means that for the application in the first scenario described above, the model could potentially remove 85.5% non-bleeding frames while preserving 95% of frames containing bleeding. For the falsepositiverate=0.0001 operating point, the Resnet50+F ensemble achieved 0.122 sensitivity, meaning that in the second application scenario, the model could potentially spot 12.2% of blood-containing frames while misdetecting blood in only 0.01% of the non-blood frames. As expected, based on the ROC curves and AUC results of the models, feature-extended models achieved higher results for most of the operating points presented.

### 5.2. Statistical Evaluation of Multiple Model Training Runs

The next experiment included a process of using multiple models for the statistical evaluation of the impact of the designed feature descriptors. For each architecture and variant (baseline and feature-extended), a set of 18 single ensembles (in a form of ×5 fold ensembles) were considered. The three ensembles that performed the worst for each variant were excluded (the exclusion rule was formulated before evaluating the models). For the 15 included single ensembles per variant (denoted as #1–15), a statistical analysis was performed by calculating the mean and standard deviation values. The analysis is presented in Table 6, Table 7, Table 8 and Table 9. In this experiment, the mean values of the ROCAUC were higher for the feature-extended models for each of the four architectures.

### 5.3. Bleeding Area Localization

**Figure 5:**
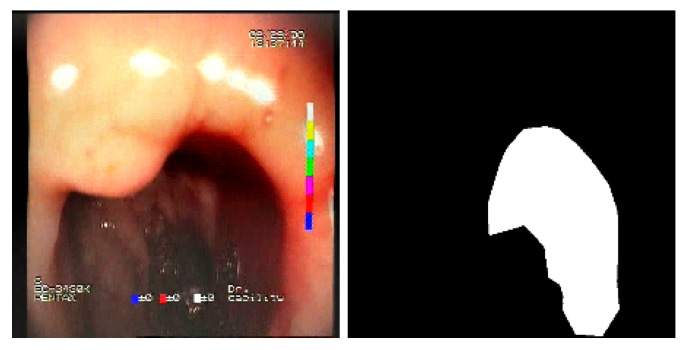
Sample test set image and the associated blood area mask.

The baseline models and the proposed feature-extended models were also evaluated in terms of providing potential explanations for classification predictions. In this case, the expected form of an explanation is the location of the bleeding areas detected by the classifier. To evaluate the quality of the localizations, a subset of the test set was used, consisting of all images for which blood area masks were available in the dataset. The subset consists of 25 test images with segmentation masks. A sample image with the associated blood area mask is presented in Figure 5. For each of the 25 test images, localizations were generated for all the evaluated models using a set of approaches and compared against the reference segmentation masks.

#### 5.3.1. Localization Accuracy Metric

The localization accuracy evaluation was performed by comparing the bounding boxes calculated over predicted locations and reference masks measured as the mean intersection over union (IoU), calculated using the standard formula:

(1) $$IoU=\frac{area(predicted\;location\;\cap \;mask)}{area(predicted\;location\;\cup \;mask)}$$

where:

predictedlocation is a set of pixels covered by the predicted bounding box region;
mask is a set of pixels covered by the bounding box of the reference location mask;
area is a function that calculates the pixel-wise area of a given region.

**Figure 6:**
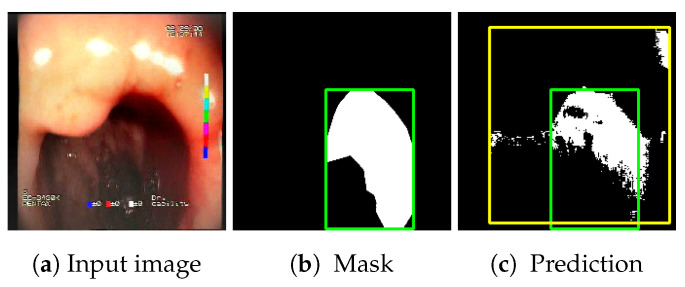
Sample prediction of blood area location acquired from the proposed feature descriptors ($F_{map}$): (**a**) input image; (**b**) reference mask with its bounding box (green); (**c**) location prediction with its bounding box (yellow) and reference mask bounding box (green). Resulting IoU is 0.327.

The choice to use bounding boxes for calculating the IoU instead of pixel-wise IoU calculation is motivated by the fact that some of the localization methods applied for the models result only in rough location areas with a fairly small resolution (e.g., 13 × 13); hence, pixel-wise evaluation would not be reliable. Bounding boxes are less vulnerable to the low resolution of the localization; therefore, they enable unified evaluation of all of the presented localization methods. An example of bounding boxes for a reference mask and location prediction is presented in Figure 6.

For each variant evaluated, the mean IoU was calculated on all images in the test set and for three × five models of the given variant, which were the three models that performed the best in each of the five cross-validation folds. For each of the variants, 15 models were evaluated in terms of localization accuracy.

#### 5.3.2. Tested Approaches

**Figure 7:**
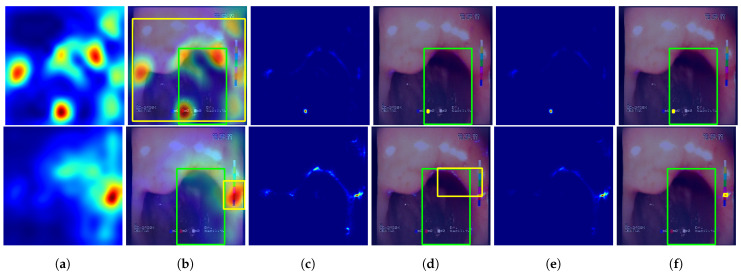
Sample predictions of the evaluated localization methods. First row—VGG19 #1 (single fold) model results, second row—VGG19+F #1 (single fold) results. Columns: (**a**) non-postprocessed GC output; (**b**) non-postprocessed GC output visualized over original image, with yellow rectangle presenting the bounding box of final GC result (postprocessed) and green rectangle presenting the bounding box of the reference mask; (**c**) non-postprocessed GP output; (**d**) non-postprocessed GP output visualized over original image with bounding boxes; (**e**) non-postprocessed GGC output; (**f**) non-postprocessed GGC output visualized over original image with bounding boxes.

**Figure 8:**
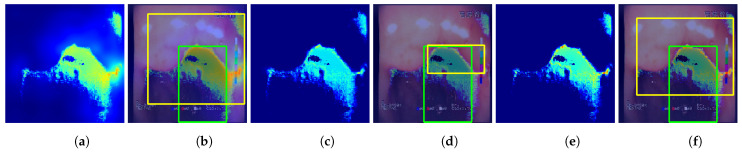
Sample predictions of the localization methods utilizing the $F_{map}$ for the VGG19+F #1 model (single fold). Columns: (**a**) non-postprocessed $GC+F_{map}$ output; (**b**) non-postprocessed $GC+F_{map}$ output visualized on original image with bounding boxes; (**c**) non-postprocessed $GP+F_{map}$ output; (**d**) non-postprocessed $GP+F_{map}$ output visualized on original image with bounding boxes; (**e**) non-postprocessed $GGC+F_{map}$ output; (**f**) non-postprocessed $GGC+F_{map}$ output visualized over original image with bounding boxes.

The following methods for generating location maps from the base and feature-extended models were used and evaluated against reference masks and respective bounding boxes. Sample results for the methods are presented in Figure 7 and Figure 8.

1. GC—Grad-Cam method [46], postprocessed using function *p* that performs normalization of the activation map to 0–1 range and thresholding at 0.5 value.
2. GP—Guided Backpropagation method [47], postprocessed with *p*.
3. GGC—Guided Grad-Cam method [48], postprocessed with *p*.
4. $F_{map}$—location map generated using the proposed features, created by averaging activations of features F1 to F7, postprocessed with *p*. This method of localization is entirely based on the designed feature descriptors and, in contrast to the GC, GP, and GGC methods, it does not involve inference of the neural network.
5. $GC+F_{map}$—proposed combination of the GC and $F_{map}$ methods by averaging the 0-1 normalized results of both methods, with the final result postprocessed with *p*.
6. $GP+F_{map}$—proposed combination of the GP and $F_{map}$ methods by averaging the 0-1 normalized results of both methods, with the final result postprocessed with *p*.
7. $GGC+F_{map}$—proposed combination of the GGC and $F_{map}$ methods by averaging the 0-1 normalized results of both methods, with the final result postprocessed with *p*.

#### 5.3.3. Localization Results

The presented localization methods were evaluated for the VGG19, Resnet50, and Inception neural network architectures for both the base and feature-extended model variants. The Resnet152 architecture was excluded due to its incompatibility with the available software libraries for the GC, GP, and GCC methods. In contrast to the evaluations presented in the previous sections, where various configurations of model ensembles were used, in this section, single models (each single model is a single convolutional neural network trained for one of the folds of the five-fold cross-validation) are evaluated separately, and the results are averaged over all single models. An evaluation is conducted for the top three single models for each of the five folds of the cross-validation training, resulting in a total of 15 models per architecture and per variant (baseline or feature-extended). The results are presented in Table 10.

Feature-extended models achieved higher results for GP localizations, while for GC and GGC localization, baseline models performed better. However, combining location predictions with $F_{map}$ improved the results for the three methods. Additionally, the $F_{map}$ localization itself achieved a mean IoU = 0.517, which is significantly higher than the GC, GP, and GCC methods for any of the models evaluated. Finally, the highest mean IoU value, equal to 0.529, was acquired for the $GC+F_{map}$ method using the VGG19+F models.

### 5.4. Processing Performance

In the final part of the experiments, the processing performance of the models was evaluated in terms of model inference time. Baseline and feature-extended model variants were evaluated for the four architectures tested. The performance was evaluated for single models by calculating the mean processing time of a single image on all images from the test set (9361 images with a resolution of 720 × 576). To enable performance measurements, a dedicated mechanism was implemented that allowed measurements of the processing time of consecutive stages of the prediction process. Several processing stages, described in Table 11, were identified and considered in the measurements.

Performance tests were conducted on a system equipped with a Ryzen Threadripper 1920X CPU (12-core/24-thread) 4.0 GHz, 64 GB RAM, Titan Xp GPU. Tests were executed separately for two variants: (1) using the CPU only, and (2) using the GPU for model prediction and the CPU for the remaining part of the processing. The results are presented in Table 12 (CPU-only variant) and Table 13 (CPU+GPU variant).

#### Performance Results

The necessity of extracting additional features obviously increases the processing time and therefore reduces the processing performance of the bleeding classification models using the feature-extended variant. The additional time is an approximately fixed-size overhead that needs to be applied once for a given input image. Hence, when more neural network models are included in a considered algorithm (e.g., 5 models of a single 5-fold cross-validation training, or 15 models of the 3× ensemble of the 5-fold models), the relative impact of the overhead on the total processing time drops. The relative impact is also clearly lower for a CPU-only system. The evaluation of the relative impact of the proposed features on the processing time for different model and system variants is presented in Table 14. It can be concluded that accuracy gains achieved using the feature-extended models justify the introduced computational overhead, especially for ensembled models.

The presented results reflect only the inference time of the algorithms. The model training time is also affected by the fixed overhead introduced by the features. However, the relative overhead is smaller during training since model prediction has to be extended with a time-consuming backward step of the backpropagation algorithm, which is obviously necessary for both the baseline and feature-extended models. Moreover, since model training is performed on long sequences of training data, feature calculation and model training steps can be easily and effectively parallelized by pipelining when using a CPU+GPU system.

Finally, the overhead presented by the proposed features is, however, not inherent. Although optimization of feature computation algorithms was not investigated in this work, the actual processing performance of the feature descriptors can be significantly improved in several aspects. For example, some of the image processing operations could be reused between the descriptors. In addition, the feature descriptors can be reimplemented using GPU programming frameworks (e.g., OpenCL, Nvidia CUDA), especially for the image processing operations used in the descriptors, for which efficient GPU implementations are available (e.g., Sobel operator, morphological operations, blur, image normalization, and other matrix operations). Therefore, the extension of CNNs in the form of the proposed feature descriptors offers great potential for improving processing performance.

## 6. Discussion

The presented work investigated the possibility of extending convolutional neural networks with a set of proposed visual features to improve accuracy for the task of endoscopic bleeding detection in color images. A methodical approach was used to theoretically define appropriate visual features and implement computer-vision-based descriptors to capture the respective features. This led to the development of valuable, high-level visual features of endoscopic bleeding. In addition, a simple scheme for combining the features with convolutional neural networks was presented; hence, the proposed features can potentially be applied along with other modifications and improvements of neural network architectures or training algorithms to achieve additional classification accuracy gains. Finally, experiments were conducted to evaluate the actual performance of the feature-extended neural networks on a representative dataset of endoscopic images.

As a result of the study, an efficient and accurate endoscopic bleeding detection algorithm was developed, achieving an ROC AUC of 0.965 in an ensembled configuration. A set of experiments confirmed the positive impact of the proposed features on the ROC AUC results for three evaluated architectures: VGG19, Resnet50, and Resnet152, and in the case of a larger sample of trained models, also for Inception v3. As well as achieving an improvement in model accuracy in terms of the ROC AUC, advantages in other accuracy metrics were also presented, including F-score metrics and the performance in high-sensitivity and high-specificity operating points. The study also evaluated the ability of the models to localize the blood area in a weakly supervised setting. A potential improvement from the feature extension was demonstrated for one of the localization methods. Furthermore, the proposed visual feature descriptors were found to be accurate predictors of bleeding localization themselves.

The accuracy results acquired in the presented study, even though it was not oriented toward optimizing accuracy but instead focused on evaluating the impact of the proposed features, are competitive in regard to the methods presented in the literature. Although several studies reported significantly higher ROC AUC values, as presented in Table 15, the direct comparison of methods is, however, difficult due to differences in the datasets used, the images sources (WC or traditional endoscopy), or the actual pathology detected (bleeding, active bleeding, angioectasia). Moreover, all of the listed approaches use private datasets, which are therefore not possible to review. The descriptions of the datasets often lack essential information, e.g., the number of included unique bleeding cases for which the positive images were extracted. A large, high-quality dataset was utilized by Kim et al. [35], which justifies the high accuracy of the method. For other studies, in turn, the size of the dataset is very low. High-accuracy results reported on small datasets are often not validated reliably enough.

The proposed method of extending deep learning models with additional visual features can be facilitated to create new or improve the accuracy of existing bleeding detection algorithms. Such methods have important clinical applications, as they can be used to develop real-time assistant applications that would support physicians in traditional endoscopy by observing the image displayed by the device as a second reader, notifying them about detected occurrences of blood and therefore reducing the risk of overlooking such lesions. In the area of wireless capsule endoscopy, where long video recordings are reviewed by the physician after examination in a time-consuming and labor-intensive process, accurate automatic detection of blood occurrences is also highly demanded. The approach proposed in the study can also be used to shorten capsule endoscopy videos and therefore reduce the physician’s time required to review examinations, although the best results would be obtained when combined with algorithms dedicated to detecting other pathologies of the gastrointestinal tract.

## Figures and Tables

**Table 1:** Definitions of the nine proposed visual features of endoscopic bleeding. For each image, a blue annotation is presented, which illustrates the area of the image where the feature is hypothetically present.

Id	Image	Feature Area	Definition
F1	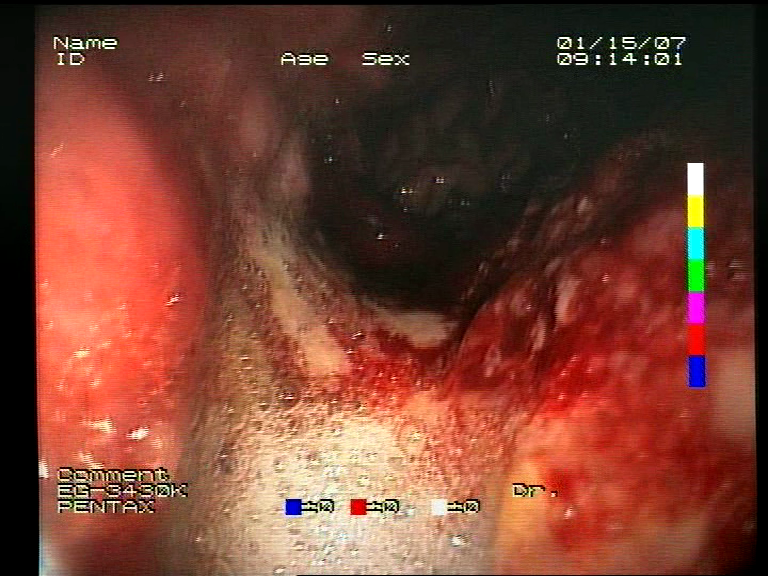	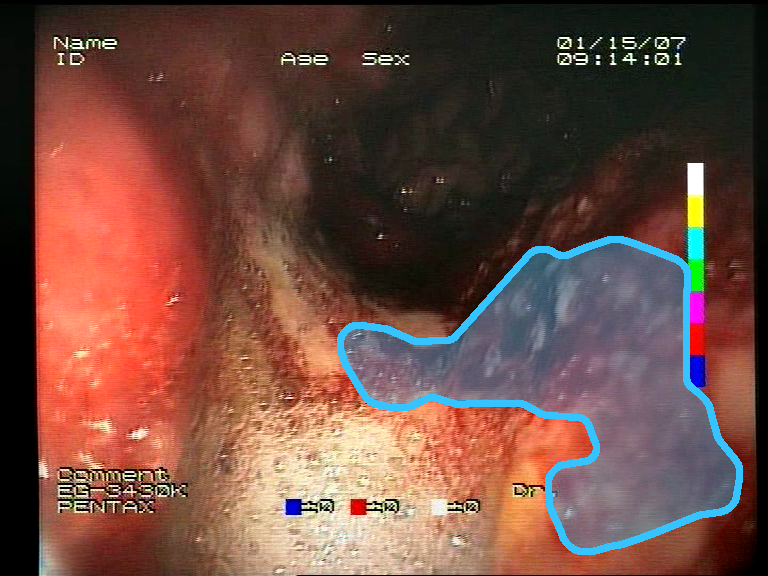	**Blood color region**—Most of the bleeding cases appear as regions of characteristic shades of red color typical of intensive bleeding or fresh blood. Therefore, color information is the strongest clue to detect bleeding. Blood color can be defined as a narrow range in a certain color space that is often present in bleeding regions and, at the same time, rarely occurs in non-blood images. The detected area must be of significant size, exceeding approximately 1% of the clearly visible part of the image.
F2	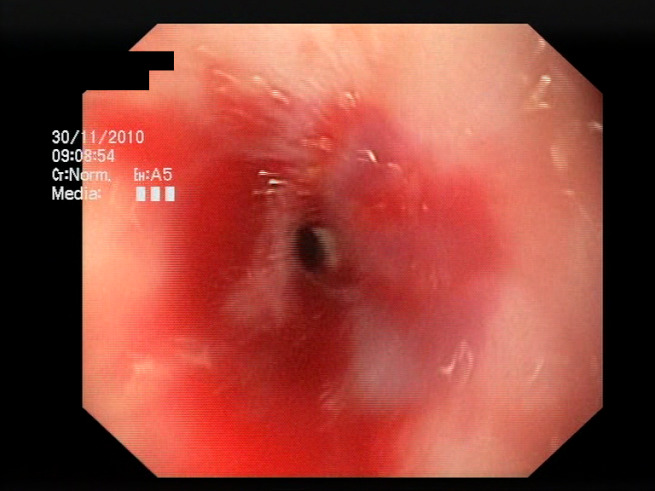	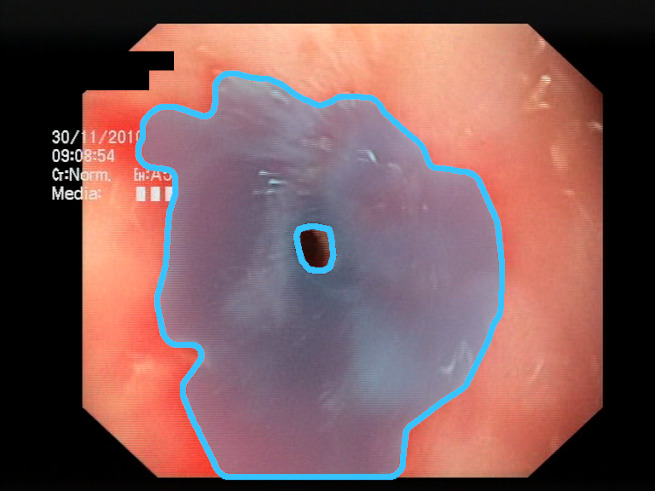	**Blood color region with a smooth surface**—In the case where the blood region is present, the occurrence of a significant amount of blood is probable. In that case, blood can fully cover the surface of the organ. This results in the appearance of a compact, smooth blood surface. The feature is considered present if at least one of the regions detected by the blood region feature has a smooth surface.
F3	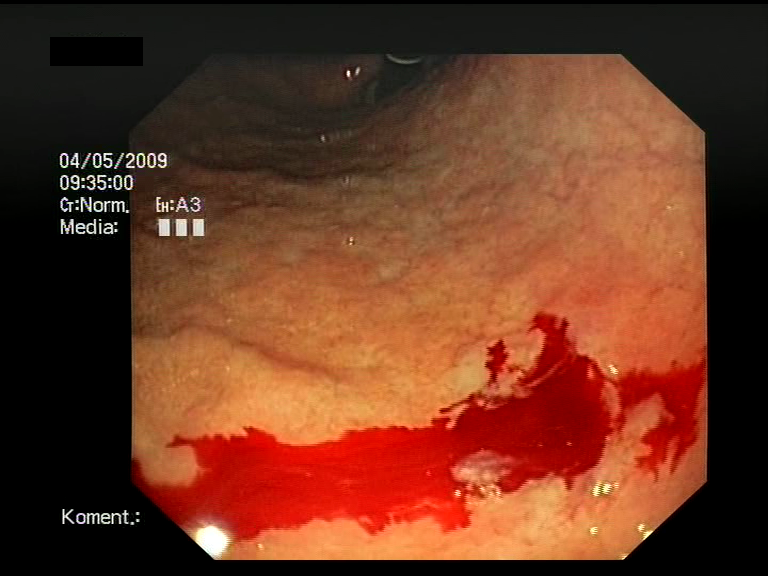	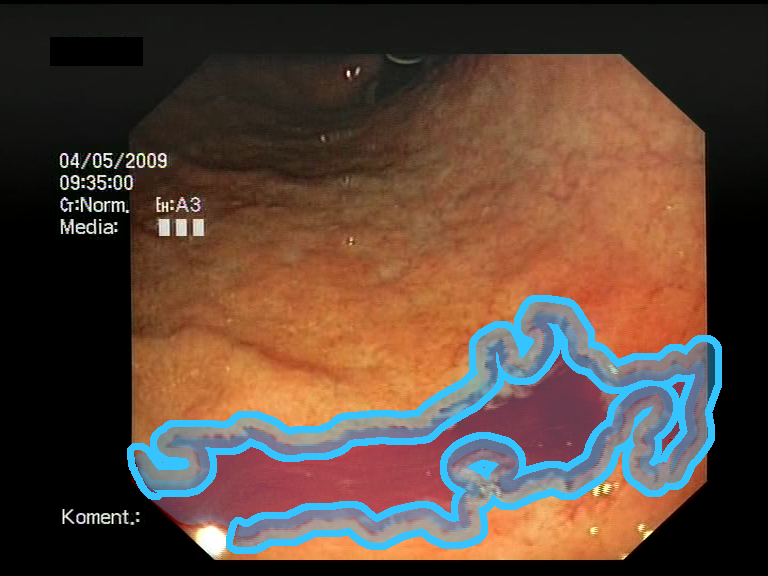	**The blood region has a clear boundary**—A fairly common characteristic of blood regions is a clear and sharp boundary separating the blood area from the normal tissue. The feature is considered present if at least a certain part of the blood region’s boundary has a clear edge separating it from the adjacent area.
F4	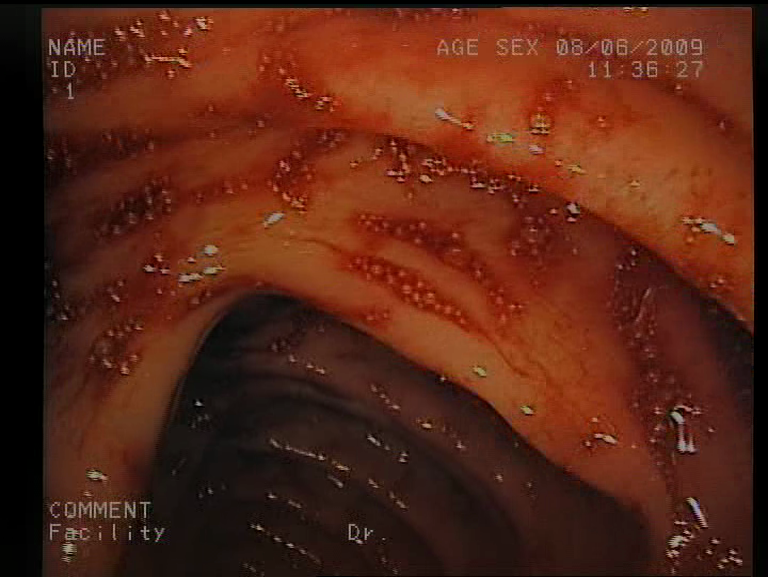	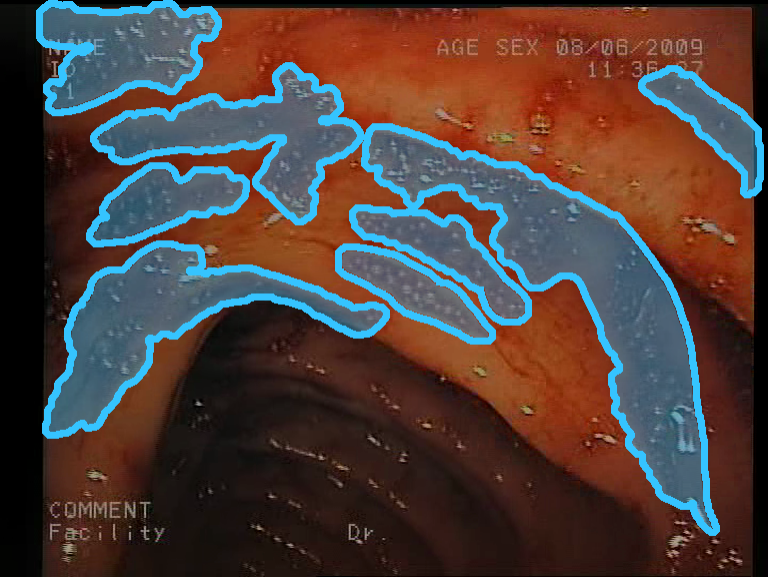	**The red color region**—Some of the bleeding cases appear as regions of a shade of red, which is less typical for blood and also appears commonly in non-blood images. To capture these cases, the feature is supposed to identify red regions similarly to the blood region feature, but with a wider range of shades of red being accepted.
F5	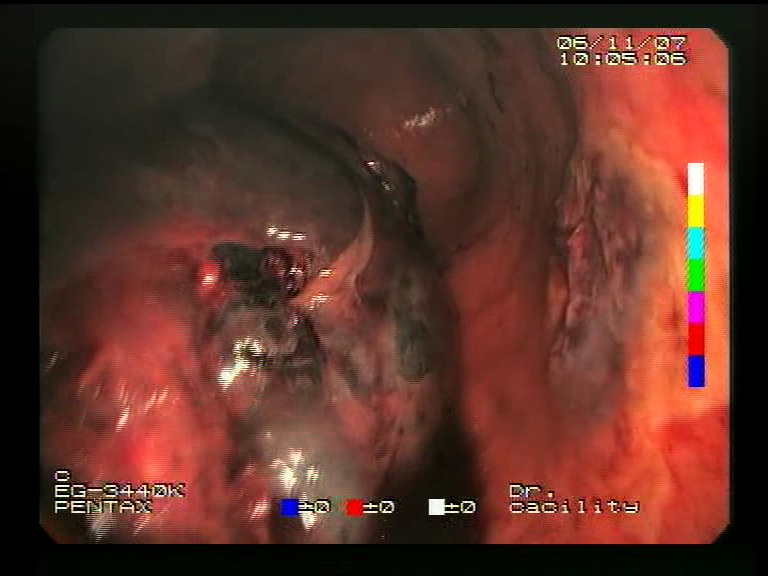	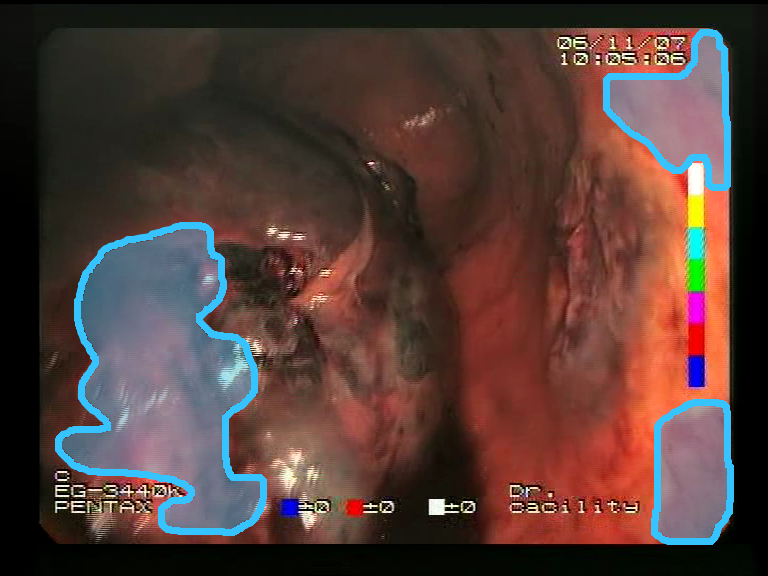	**Red color region with a smooth surface**—The feature is related to the previous feature and has a similar motivation as the smooth blood region feature (F2). The parameters of the expected descriptor might, however, be changed as a result of tuning towards specificity of red regions.
F6	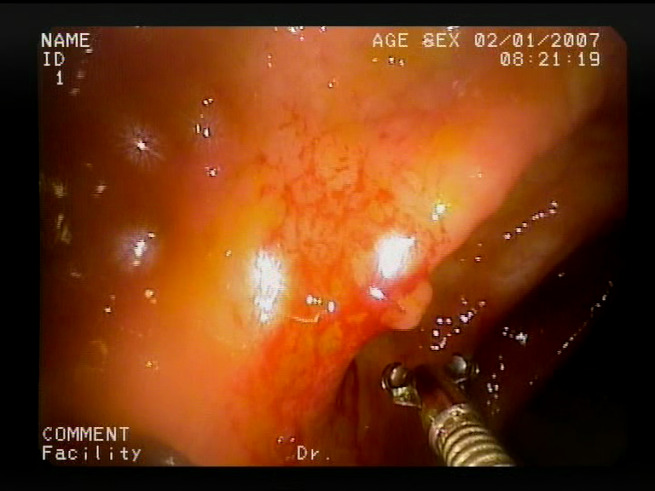	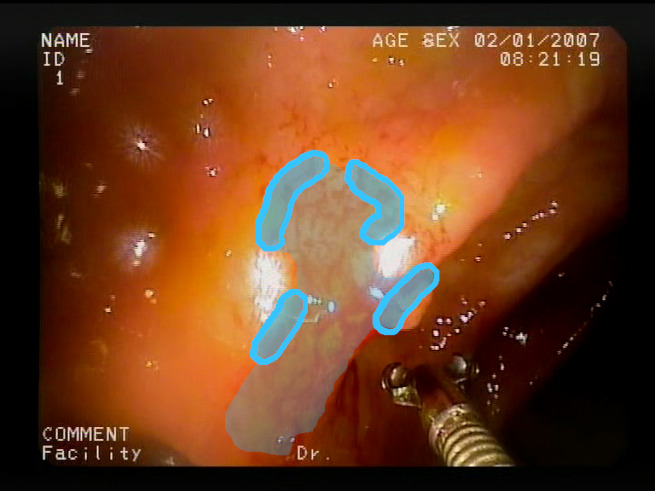	**The red region has a clear boundary**—The feature is the equivalent of a clear boundary feature (F3) for the red color region feature. Similarly to the previous feature, some descriptor parameters may differ due to adjustments made to the red regions.
F7	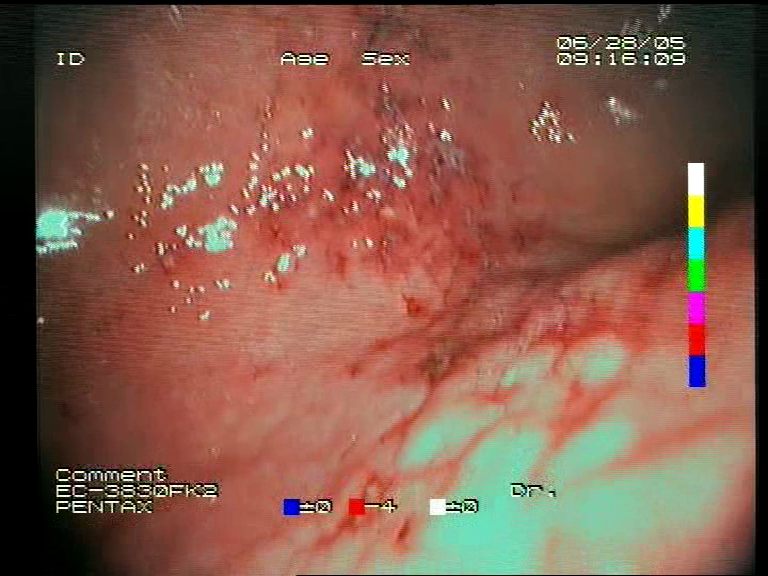	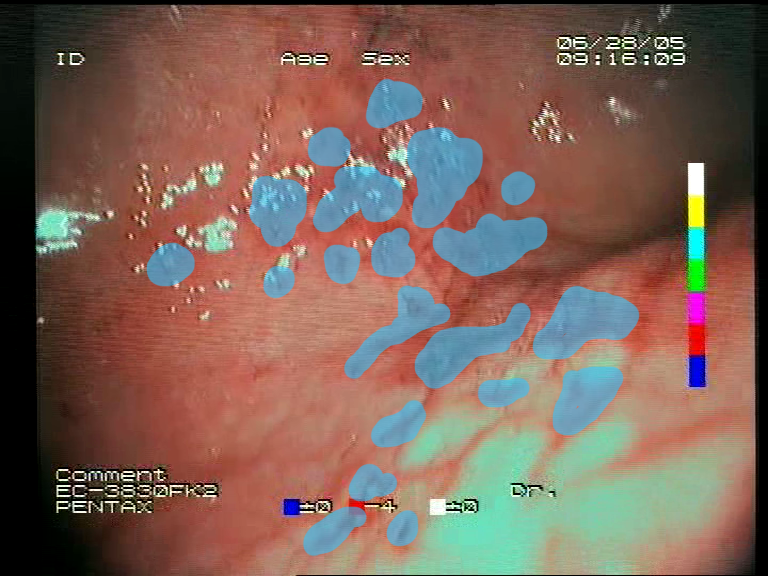	**Dispersed blood color**—Small amounts of blood can appear as small, spread areas, spots, or thin blood streams. In contrast to the blood region feature, large regions are ignored here, and the color is validated on a pixel level. However, some larger areas of blood contaminated by other fluids or bubbles can also be detected because they do not form continuous areas of blood color. Again, a narrow range of red color needs to be defined to describe blood.
F8	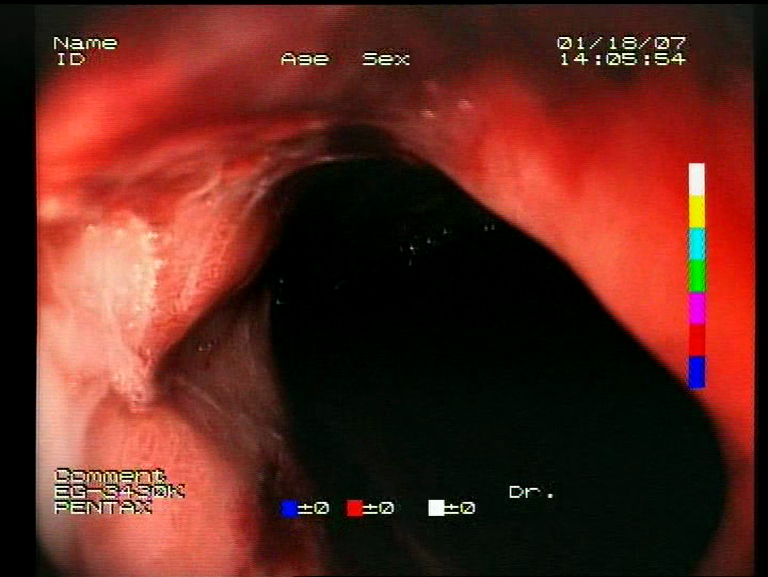	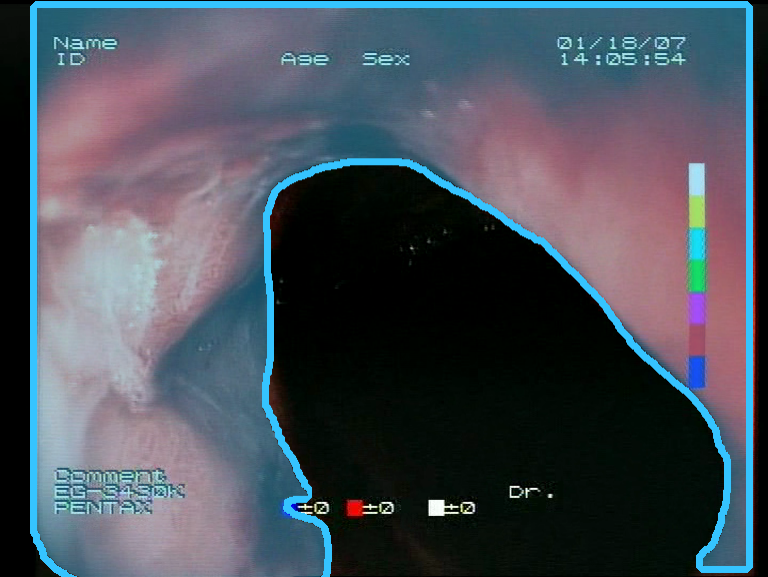	**Red color domination**—An additional clue for analyzing the image is provided by the assessment of a dominant color in the image. The feature is considered present if the dominant color of the image is red; that is, the red color covers more than 50% of the image, excluding unclear parts of the image resulting from highlights or darkness. The red color domination is considered to be an excess of the red component in the RGB color space, allowing a wide range of shades of red.
F9	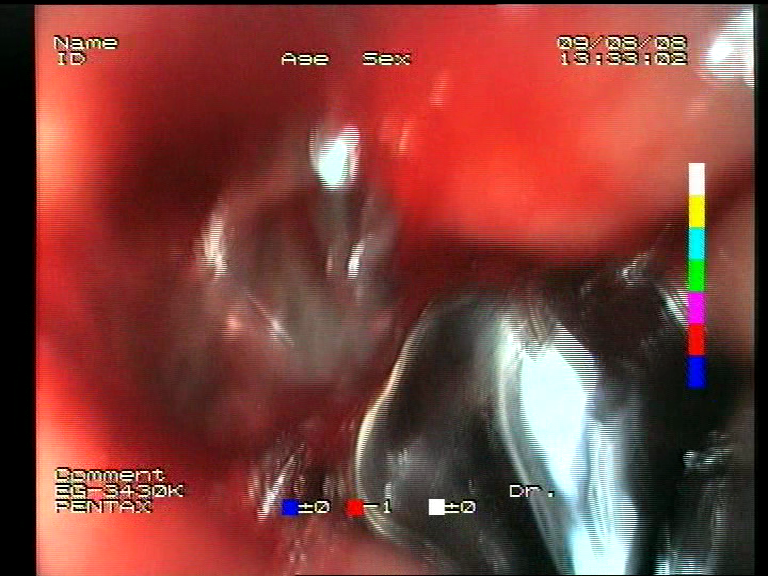	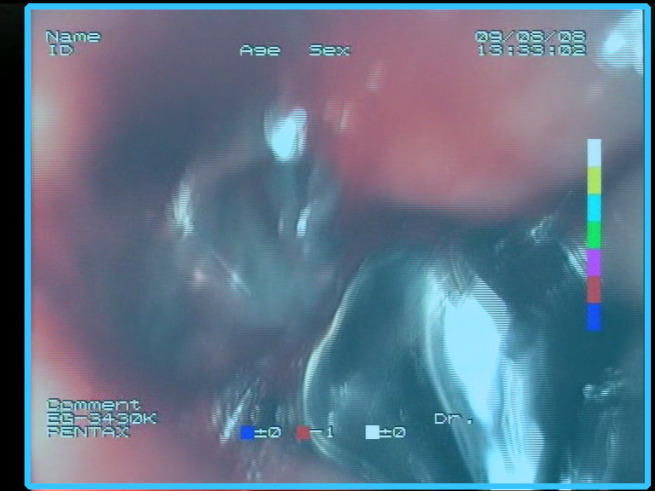	**Image is blurred**—Blurriness significantly affects image analysis, hindering the identification of bleeding areas. It also affects the evaluation of the remaining features. Therefore, the blurriness of the image is also evaluated as a feature. Blurriness can be evaluated by detecting edges in the image and measuring the variance in the colors in the image. High variance with a low number of edges is an indication of possible blurriness.

**Table 2:** Statistics of the ERS dataset used in the experiments, including number of endoscopic examinations videos, number of samples extracted from the videos, and total images available in all the extracted samples.

Set	Class	Endoscopic Examinations	Endoscopic Samples	Endoscopic Images
Training	blood	56	58	22,545
	non-blood	226	331	17,212
Test	blood	19	19	6912
	non-blood	76	113	7461
Total	blood	75	77	29,457
	non-blood	302	444	24,673

**Table 3:** Classification accuracy metrics used in the evaluation.

Metric	hlDefinition
ROCAUC	area under the receiver operating characteristic curve
F1	$2\cdot \frac{precision\cdot recall}{precision+recall}$
F2	$5\cdot \frac{precision\cdot recall}{4\cdot precision+recall}$
sensitivity	$\frac{TP}{TP+FN}$
specificity	$\frac{TN}{TN+FP}$
precision	$\frac{TP}{TP+FP}$
accuracy	$\frac{TP+TN}{TP+FP+TN+FN}$
sens@0.01	sensitivity calculated for the classification threshold resulting in falsepositiverate value equal to 0.01
sens@0.001	sensitivity for falsepositiverate equal to 0.001
sens@0.0001	sensitivity for falsepositiverate equal to 0.0001
spec@0.95	specificity for sensitivity equal to 0.95
sens@0.99	specificity for sensitivity equal to 0.99
sens@0.999	specificity for sensitivity equal to 0.999

**Table 4:** Additional terms used in the evaluation metrics.

Metric	Definition
TP	number of correctly classified blood cases, calculated at 0.5 classification threshold
FP	number of incorrectly classified blood cases at the 0.5 threshold
TN	number of correctly classified normal cases at the 0.5 threshold
FN	number of incorrectly classified normal cases at the 0.5 threshold
recall	equivalent to sensitivity
truepositiverate	equivalent to sensitivity
falsepositiverate	1−specificity

**Table 5:** The best single ensemble (denoted as #1—ensembles of 5 folds in each case) results for baseline (base) and feature-extended variants (+F). For the VGG19 and Resnet152 architectures, the feature-extended ensembles reached a higher ROC AUC. Overall accuracy was also better, although the advantage is not clear. For Resnet50, the feature-extended ensemble reached a significantly higher ROC AUC and also had a clearly higher overall accuracy. In the case of the Inception architecture, the baseline ensemble achieved a higher ROC AUC than the feature-extended ensemble, and the overall accuracy was higher for the baseline ensemble.

Metric	VGG19		Resnet50		Resnet152		Inception	
	**×5 Folds #1**		**×5 Folds #1**		**×5 Folds #1**		**×5 Folds #1**	
	**Base**	**+F**	**Base**	**+F**	**Base**	**+F**	**Base**	**+F**
ROCAUC	0.891	**0.959**	0.918	**0.963**	0.929	**0.936**	**0.954**	0.942
F1	**0.762**	0.755	0.684	**0.865**	**0.758**	0.720	**0.797**	0.776
F2	0.785	**0.854**	0.713	**0.888**	0.751	**0.801**	**0.863**	0.786
sensitivity	0.801	**0.936**	0.734	**0.904**	0.746	**0.867**	**0.914**	0.794
specificity	**0.923**	0.862	0.895	**0.953**	**0.943**	0.862	0.903	**0.936**
precision	**0.726**	0.633	0.640	**0.829**	**0.770**	0.615	0.707	**0.759**
accuracy	**0.898**	0.877	0.862	**0.943**	**0.903**	0.863	0.906	**0.907**
sens@0.01	0.561	**0.622**	0.513	**0.667**	**0.591**	0.522	0.583	**0.606**
sens@0.001	**0.341**	0.226	0.138	**0.251**	0.131	**0.171**	0.184	**0.198**
sens@0.0001	**0.068**	0.010	0.025	**0.122**	0.005	**0.094**	**0.020**	0.004
spec@0.95	0.286	**0.772**	0.649	**0.855**	0.568	**0.775**	**0.764**	0.743
spec@0.99	0.088	**0.295**	**0.254**	0.215	**0.331**	0.155	0.191	**0.327**
spec@0.999	0.008	**0.028**	**0.007**	0.003	0.012	**0.017**	0.034	**0.063**

**Table 6:** Mean metric values calculated for 15 base VGG19 and 15 feature-extended single ensembles.

Metric	VGG19 ×5 Folds #1–15		VGG19+F ×5 Folds #1–15	
	**Mean**	**STD**	**Mean**	**STD**
ROCAUC	0.923	0.018	**0.962**	0.004
F1	0.778	0.014	**0.801**	0.022
F2	0.783	0.014	**0.870**	0.009
sensitivity	0.787	0.018	**0.924**	0.006
specificity	**0.940**	0.008	0.902	0.018
precision	**0.769**	0.024	0.708	0.035
accuracy	**0.909**	0.007	0.906	0.013
sens@0.01	0.579	0.039	**0.627**	0.027
sens@0.001	**0.315**	0.038	0.271	0.053
sens@0.0001	**0.079**	0.041	0.038	0.073
spec@0.95	0.566	0.171	**0.815**	0.037
spec@0.99	0.146	0.050	**0.323**	0.053
spec@0.999	0.027	0.022	**0.053**	0.039

**Table 7:** Mean metric values calculated for 15 base Resnet50 and 15 feature-extended single ensembles.

Metric	Resnet50 ×5 Folds #1–15		Resnet50+F ×5 Folds #1–15	
	**Mean**	**STD**	**Mean**	**STD**
ROCAUC	0.913	0.012	**0.953**	0.007
F1	0.705	0.033	**0.813**	0.030
F2	0.751	0.021	**0.824**	0.032
sensitivity	0.786	0.031	**0.833**	0.049
specificity	0.886	0.032	**0.944**	0.028
precision	0.643	0.061	**0.800**	0.063
accuracy	0.865	0.023	**0.922**	0.017
sens@0.01	0.422	0.040	**0.582**	0.069
sens@0.001	0.141	0.066	**0.195**	0.062
sens@0.0001	0.026	0.032	**0.062**	0.039
spec@0.95	0.588	0.078	**0.796**	0.048
spec@0.99	**0.211**	0.088	0.182	0.085
spec@0.999	**0.013**	0.012	0.009	0.012

**Table 8:** Mean metric values calculated for 15 base Resnet152 and 15 feature-extended single ensembles.

Metric	Resnet152 ×5 Folds #1–15		Resnet152+F ×5 Folds #1–15	
	**Mean**	**STD**	**Mean**	**STD**
ROCAUC	0.935	0.013	**0.946**	0.007
F1	0.760	0.027	**0.787**	0.029
F2	0.785	0.026	**0.813**	0.034
sensitivity	0.803	0.038	**0.833**	0.055
specificity	0.920	0.024	**0.927**	0.029
precision	0.724	0.051	**0.753**	0.064
accuracy	0.896	0.016	**0.908**	0.017
sens@0.01	0.508	0.052	**0.616**	0.058
sens@0.001	0.156	0.071	**0.193**	0.078
sens@0.0001	0.044	0.043	**0.049**	0.059
spec@0.95	0.641	0.142	**0.747**	0.062
spec@0.99	**0.257**	0.076	0.147	0.041
spec@0.999	**0.014**	0.015	0.008	0.014

**Table 9:** Mean metric values calculated for 15 base Inception-v3 and 15 feature-extended single ensembles.

Metric	Inception ×5 Folds #1–15		Inception+F ×5 Folds #1–15	
	**Mean**	**STD**	**Mean**	**STD**
ROCAUC	0.947	0.007	**0.956**	0.010
F1	0.782	0.025	**0.806**	0.028
F2	0.784	0.042	**0.817**	0.033
sensitivity	0.788	0.064	**0.825**	0.043
specificity	0.942	0.027	**0.943**	0.015
precision	0.785	0.067	**0.789**	0.044
accuracy	0.910	0.014	**0.919**	0.012
sens@0.01	0.540	0.054	**0.618**	0.057
sens@0.001	0.185	0.074	**0.226**	0.076
sens@0.0001	0.048	0.049	**0.059**	0.041
spec@0.95	0.734	0.066	**0.809**	0.084
spec@0.99	0.294	0.055	**0.312**	0.064
spec@0.999	0.032	0.034	**0.043**	0.039

**Table 10:** Mean IoU values acquired for bleeding area localization for each architecture, variant (baseline—“base”; feature-extended—“+F”) and localization method, averaged over all 25 images from the test set and 15 (top 3 × 5 folds) models. “-” indicates that the given method was not evaluated. Mean IoU value of $F_{map}$ localization map itself achieved the value of 0.517.

Method	VGG19		Resnet50		Inception	
	**Base**	**+F**	**Base**	**+F**	**Base**	**+F**
GC	**0.423**	0.396	0.369	0.369	**0.381**	0.334
$GC+F_{map}$	-	**0.529**	-	**0.497**	-	**0.485**
GP	0.203	0.266	0.223	0.231	0.176	**0.249**
$GP+F_{map}$	-	**0.387**	-	**0.394**	-	**0.398**
GGC	**0.154**	0.148	**0.182**	0.179	0.156	0.217
$GGC+F_{map}$	-	**0.298**	-	**0.340**	-	**0.374**

**Table 11:** Processing stages of the complete algorithm evaluated in the performance tests.

Stage	Definition
preprocess	Preparation of the input image for the actual processing, including deinterlacing and resizing using cubic interpolation.
roi	Additional preprocessing step required by the proposed feature descriptors, including detection of the regionofinterest area of the image presenting the actual organ (that is, excluding black border and overlay information printed in the image by the endoscopic device).
F1–F9	Calculation of each of the F1–F9 feature planes using the corresponding feature descriptor.
featurestotal	Total processing time of all feature descriptors (excluding roi detection).
predict	Inference process of the neural network model.
totaltime	Total processing time of a single endoscopic image.

**Table 12:** Single model inference mean processing time in milliseconds for a CPU-only system. “-” denotes stages that are not present in the baseline (“base”) variants.

Stage	VGG19		Resnet50		Resnet152		Inception	
	**Base**	**+F**	**Base**	**+F**	**Base**	**+F**	**Base**	**+F**
preprocess	**1.14**	1.42	**1.18**	1.43	**1.17**	1.43	**1.28**	1.45
roi	-	13.75	-	14.18	-	14.15	-	14.29
F1	-	3.95	-	4.04	-	4.36	-	4.26
F2	-	1.74	-	1.78	-	1.82	-	1.80
F3	-	1.83	-	1.84	-	1.89	-	1.86
F4	-	3.82	-	3.83	-	3.91	-	4.01
F5	-	1.71	-	1.72	-	1.78	-	1.72
F6	-	1.82	-	1.81	-	1.87	-	1.84
F7	-	0.13	-	0.14	-	0.15	-	0.14
F8	-	0.77	-	0.78	-	0.80	-	0.78
F9	-	1.34	-	1.34	-	1.39	-	1.34
featurestotal	-	31.30	-	31.90	-	32.56	-	32.46
predict	**181.84**	183.18	**87.82**	89.52	**231.13**	233.41	**57.82**	57.94
totaltime	**183.25**	217.71	**89.31**	124.90	**232.60**	269.29	**59.41**	93.86

**Table 13:** Single model inference mean processing time in miliseconds for a CPU+GPU system, where predict stage is performed using GPU, while the remaining stages are performed on CPU. “-” denotes stages that are not present in the baseline (“base”) variants.

Stage	VGG19		Resnet50		Resnet152		Inception	
	**Base**	**+F**	**Base**	**+F**	**Base**	**+F**	**Base**	**+F**
preprocess	**1.35**	1.44	**1.30**	1.45	**1.22**	1.48	**1.30**	1.58
roi	-	13.52	-	13.90	-	13.83	-	14.11
featurestotal	-	30.68	-	31.47	-	31.53	-	31.73
predict	**9.03**	11.34	**19.13**	20.75	**51.30**	55.30	**29.74**	31.34
totaltime	**10.67**	45.16	**20.73**	55.47	**52.82**	90.07	**31.34**	66.52

**Table 14:** Relative overhead in inference time of feature-extended models in comparison to baseline models for different model and system variants.

Model Variant	System Variant	VGG19	Resnet50	Resnet152	Inception
single model	cpu	+18.8%	+39.9%	+15.8%	+58.0%
×5 fold		+5.7%	+12.5%	+5.0%	+16.4%
ensemble ×3		+2.4%	+5.4%	+2.3%	+5.6%
single model	cpu+gpu	+323.1%	+167.6%	+70.5%	+112.3%
×5 fold		+120.0%	+55.3%	+25.5%	+36.1%
ensemble ×3		+57.6%	+24.2%	+13.7%	+15.7%

**Table 15:** Comparison of the presented study results of ROC AUC values reported in the literature.

Method	Year	Dataset	Total Positive (Blood) Images	Image Source	Lesion Type	ROC AUC
Li et al. [23]	2017	Private dataset	185	WCE	Bleeding	0.991
Tsuboi et al. [24]	2019	Private dataset	2725	WCE	Angioectasia	0.998
Aoki et al. [25]	2021	Private dataset	2237 + 29 videos	WCE	Angioectasia	0.871
Kim et al. [35]	2021	Private dataset	164,713	WCE	Bleeding	0.922–0.998
This study	2023	ERS [45]	29,457	Traditional endoscopy	Bleeding	0.965

## Data Availability

Publicly available datasets were analyzed in this study. These data can be found here: https://cvlab.eti.pg.gda.pl/pl/publications/endoscopy-dataset, (accessed on 7 December 2023) .
